# FGFBP2 p.T186S mutation in tumor associated fibroblasts supports progression of pancreatic cancer through autocrine and paracrine mechanisms

**DOI:** 10.1186/s12967-026-09137-3

**Published:** 2026-11-10

**Authors:** Xing Wu, Bolin Zhang, Yuhang Shen, Yijun Chen, Bogusz Trojanowicz, Yoshiaki Sunami, Jörg Kleeff

**Affiliations:** 1 Department of Visceral, Vascular and Endocrine Surgery, Martin-Luther- University Halle-Wittenberg, University Medical Center Halle, Ernst-Grube-Str. 40, 06120 Halle (Saale), Germany; 2 Department of General Surgery, The Second Hospital of Anhui Medical University, Hefei, Anhui China; 3 Department of Thyroid Surgery, The Affiliated Hospital of Putian University, Putian, Fujian China

**Keywords:** Pancreatic cancer, FGFBP2, Tumor microenvironment, CAFs, Single-cell RNA sequencing

## Abstract

**Background:**

Pancreatic cancer is one of the most aggressive human tumors with an extremely poor prognosis. The tumor microenvironment is characterized by a complex network of cellular interactions, in which cancer-associated fibroblasts (CAFs) play a pivotal role in the initiation and progression of the disease. Previously identified somatic cancer-associated fibroblast (CAF) mutations may have functional consequences thereby affecting pancreatic cancer biology. One of the identified mutations of the fibroblast growth factor binding protein 2 gene (*FGFBP2*) causes replacement from threonine to serine (p.T186S). The aim of this study was to investigate how this mutation may affect fibroblast function, the tumor microenvironment and cancer progression.

**Methods:**

FGFBP2 p.T186S mutation harboring fibroblasts (FGFBP2^MUT^) and wild-type fibroblasts (FGFBP2^WT^) were analyzed for activation markers, viable cell numbers, and migration. PANC-1 pancreatic cancer cells were incubated with conditioned media from FGFBP2^MUT^ and FGFBP2^WT^ fibroblasts to assess viable cell numbers and signaling pathway activation. RNA sequencing was performed to identify differentially expressed genes. Furthermore, publicly available single-cell RNA sequencing and spatial transcriptomic datasets were analyzed to explore downstream molecular mechanisms.

**Results:**

FGFBP2 p.T186S mutation harboring cells (FGFBP2^MUT^) displayed increased expression of activation markers and cytokines as compared to controls. FGFBP2^MUT^ cells exhibited increased viable cell numbers and migration. Further, conditioned medium derived from FGFBP2^MUT^ promoted growth of the pancreatic cancer cell line PANC-1 and resulted in increased activation of the ERK1/2 signaling pathway. RNA sequencing analysis of PANC-1 cells treated with conditioned media derived from FGFBP2^MUT^ revealed upregulation of the ETS homologous factor (*EHF*) gene. Single-cell RNA sequencing analysis showed that EHF was primarily expressed in tumor cells and regulated fibroblasts through the platelet-derived growth factor (PDGF) signaling pathway. In addition, spatial transcriptomic analysis revealed a correlation between *EHF* expression and CAF markers *ACTA2*, *POSTN*, and *COL1A1*.

**Conclusions:**

Our study shows that the FGFBP2 p.T186S point mutation promotes fibroblast activation potentially influencing tumor progression by increasing the viable cell population of pancreatic cancer cells.

**Supplementary Information:**

The online version contains supplementary material available at https://doi.org/10.1186/s12967-026-09137-3.

## Introduction

Pancreatic ductal adenocarcinoma (PDAC) is the fourth leading cause of cancer-related deaths in the USA [1]. It remains one of the most aggressive solid tumors, which is predicted to become the second leading cause of cancer-related deaths in the Western world by 2030 [2]. This dismal outcome is largely attributed to the highly complex tumor microenvironment, which plays a pivotal role in tumor progression and mediating resistance to therapy [3]. Among the various cellular components of the tumor microenvironment, cancer-associated fibroblasts (CAFs) are key contributors to the initiation and progression of pancreatic cancer [4]. Fibroblast infiltration and activation in the proximity of pancreatic cancer is a long-known phenomenon. Activated CAFs express a variety of markers, including alpha-smooth muscle actin (α-SMA). Numerous factors, including signals from tumor cells, the surrounding microenvironment, and inflammatory mediators, have been shown to contribute to CAF activation [5–7]. Moreover, activated CAFs secrete cytokines, growth factors, and extracellular matrix (ECM) proteins, which play crucial roles in promoting tumor progression and metastasis [8–10].

FGFBP2 is a member of the fibroblast growth factor binding protein family. Growth factor signaling is known to play an important role in tumorigenesis and development. Among the growth factors and receptors involved in the malignant process, fibroblast growth factors (FGF), a family of more than 20 proteins, have been described to enhance tumor growth, angiogenesis, and progression [11, 12]. FGFBP2 is constitutively secreted by Th1-type CD4-positive lymphocytes and lymphocytes with cytotoxic potential and may be involved in an essential process of cytotoxic lymphocyte-mediated immunity [13].

FGFBP2 stabilizes and enhances the bioavailability of FGF2, facilitating its interaction with FGF receptors (FGFRs) [14, 15]. Activation of FGFRs triggers downstream signaling cascades, including the ERK and AKT pathways, which regulate cell proliferation, and migration [16]. Yamanaka et al. demonstrated that FGFBP2 expression in high-grade gliomas was positively correlated with survival [17]. Early studies showed that acidic fibroblast growth factor (aFGF) and basic FGF (bFGF) are frequently overexpressed in pancreatic cancer cells and correlate with advanced tumor stage and poor patient survival [18]. Subsequent studies demonstrated that aberrant expression and fibroblast growth factor receptor 2 (FGFR2) promote pancreatic cancer cell proliferation, invasion, ERK activation, and metastatic progression, and that *FGFR2* inhibition suppresses these malignant phenotypes [19, 20]. More recently, genetic evidence revealed that FGFR2 inhibition impairs mutant KRAS–expressing pancreatic cancer and delays pancreatic tumorigenesis [21]. In addition to tumor-intrinsic *FGFR2* signaling, FGFBP1-mediated FGF22 from CAFs promote pancreatic cancer invasion [22]. Previous next-generation sequencing (NGS) studies performed on CAFs originating from pancreatic cancer patients revealed a FGFBP2 point guanine (G) to cytidine (C) mutation, causing an amino acid change from threonine to serine (FGFBP2 p.T186S), in CAFs but not in the peripheral blood DNA of a pancreatic cancer patient [23]. Since the role of this FGFBP2 mutation in pancreatic carcinogenesis is unknown, the aim of this study was to investigate how this somatic mutation in FGFBP2 affects fibroblast function in the pancreatic cancer microenvironment and how these interactions might contribute to tumor progression.

## Materials and methods

### Cell culture

SV80 cells, which were immortalized by the SV40 virus, were derived from healthy adult female skin fibroblasts. SV80 cells were purchased from CLS Cell Lines Service GmbH (Eppelheim, Germany), and PANC-1 and PaTu 8902 cells were purchased from American Type Culture Collection (Manassas, USA). SV80 cells were selected as an established immortalized human fibroblast model to investigate the functional effects of the FGFBP2 p.T186S mutation. PANC-1 was used as a well-characterized pancreatic ductal adenocarcinoma cell line, and PaTu 8902 was included to validate the reproducibility of the findings in an additional pancreatic cancer cell line. Both SV80, PaTu 8902, and PANC-1 cells were cultured in an incubator at 37 °C with 5% CO_2_ and maintained in high-glucose Dulbecco’s Modified Eagle’s Medium (DMEM) (Sigma-Aldrich, Germany) supplemented with 10% fetal bovine serum (FBS), 100 U/mL penicillin, and 100 ug/mL streptomycin.

### Plasmids and cell transfection

The mammalian Expression Vector pCMV6-AC-GFP (PS100010, OriGene, USA) served as construct for empty control plasmids or cloned FGFBP2 ORFs with (p.T186S) (CW307647) or without point mutation (RG206392). All constructs were prepared by transformation using dam-/dcm- competent E. coli cells (New England Biolabs, C2925H, USA) and plasmid extraction using the ZymoPURE - Express™ Plasmid Midiprep Kit (ZYMO RESEARCH EUROPE GMBH, D4213, Germany). The day before transfection, SV80 cells were seeded in a 24-well plate at 6 × 10^5^ cells per well. Prior to transfection, the medium was replaced with serum-free medium. Lipofectamine^®^ 3000 (Thermo Fisher Scientific, Germany) and plasmid DNA (with pT3000) were diluted in Opti-MEM^®^. At 72 h, 0.6 mg/ml neomycin selection was applied.

EHF siRNA (Silencer™ Select Pre-designed siRNA, siRNA ID: s25398, Cat. No. 4392420, Thermo Fisher Scientific, Germany) and a negative control siRNA (Cat. No. 4390843, Thermo Fisher Scientific, Germany) were transfected into PANC-1 cells using Lipofectamine^®^ 3000 Transfection Reagent according to the manufacturer’s instructions. The EHF siRNA sequences were 5′-GGACGAAGACUGGUAUAUAtt-3′ (sense) and 5′-UAUAUACCAGUCUUCGUCCat-3′ (antisense). Transfection efficiency was examined 48 h after transfection. For EHF overexpression, the human EHF expression plasmid RG207904 (OriGene, USA), containing the EHF (NM_012153) open reading frame, was used, whereas the corresponding empty vector served as the control. For EHF re-expression following knockdown, cells were first transfected with EHF siRNA and subsequently with RG207904. The s25398 target sequence is located within the EHF coding region present in RG207904, and the construct was not engineered to contain siRNA-resistance-conferring substitutions. Restoration of EHF protein expression following sequential transfection was confirmed by immunoblotting.

### Cell viability assay

Cells were seeded in a 96-well plate at a density of 3 × 10^3^ cells/ml and incubated at 37 °C with 5% CO_2_ for 24 h. At 24, 48, and 72 h, 20 µl of Thiazolyl Blue Tetrazolium Bromide (MTT; prepared by dissolving 25 mg MTT in 5 ml HBSS, Sigma-Aldrich, USA) was added to each well. The plate was then returned to the incubator for an additional 4 h, after which 100 µl of DMSO was added to each well. Absorbance was measured at 570 nm using a Sunrise Absorbance Microplate Reader.

### Migration assay

Cells (1 × 10^4^ in 200 µL serum-free medium) were seeded onto each transwell insert, which was then placed in a 24-well plate containing 500 µL of medium with 10% FBS and incubated at 37 °C with 5% CO₂ for 24 h. After incubation, the cells were fixed with 4% paraformaldehyde and stained with hematoxylin. The upper surface of the insert was cleaned, and the inserts were then examined under a microscope to assess cell motility. Images were captured from five random areas using inverted fluorescence phase contrast microscopy, and the number of cells per field was counted and analyzed using ImageJ (v.1.5.3).

### Preparation of conditioned medium

To prepare conditioned medium, 3 × 10^5^ cells were seeded with culture medium containing 10% FBS and maintained under standard conditions. After 24 h, the cells were washed with PBS and cultured with serum-free medium (0% FBS). The cells were then cultured for an additional 48 h, after which the culture medium was collected, centrifuged to remove cell debris, and stored at -80 °C until further use.

### ELISA

The concentrations of IL-6 and FGF2 in conditioned media were measured using human ELISA kits (Proteintech; Human IL-6 ELISA Kit, Cat#: KE00385; Human FGF2 ELISA Kit, Cat#: KE00129, China) according to the manufacturer’s instructions. Briefly, conditioned media were collected, and the supernatants were subjected to ELISA analysis. Cytokine concentrations were calculated based on standard curves.

### FGF2 and EGF treatment, MEK inhibition and IL-6R blockade

To investigate the effects of conditioned media (CM) and CM combined with recombinant FGF2 or EGF on ERK1/2 and AKT phosphorylation, PANC-1 cells were seeded in a medium containing 10% FBS and incubated for 24 h. The medium was then replaced with serum-free medium, and the cells were cultured for an additional 12 h. Recombinant FGF2 or EGF was added directly to the culture medium to achieve a final concentration of 10 ng/ml, and the cells were treated for 0, 5, and 20 min. For inhibition experiments, PANC-1 cells were treated with conditioned media containing the MEK inhibitor U0126 (Cell Signaling Technology, USA) at a final concentration of 10µM for 2 h. For IL-6R blockade experiments, cells were pretreated with tocilizumab at 1 µg/mL for 1 h before stimulation with conditioned medium.

### qPCR

Total RNA was extracted from cells and isolated according to manufacturer’s protocol using direct-zol™ Mini-prep Kit (Zymo research Europe GmbH, Germany). qPCR was performed using HOT FIREPol^®^ EvaGreen^®^ qPCR Mix Plus (ROX) (Solis BioDyne, Estonia). Human primers sequences are listed in Table S1.

### Western blot

Total protein was isolated using lysis buffer. Proteins were separated by SDS-PAGE and then transferred onto PVDF Blotting Membranes (GE Healtcare Life science, Great Britain). The membranes were incubated overnight at 4 °C with primary antibodies, thereafter with secondary antibodies at RT for 1 h. The antibodies used were anti-FGFBP2 (ThermoFisher, PA5-113480, 1:1000), anti-ERK1/2 (Cell Signaling Technology, #4695, 1:2000), anti-P-ERK1/2 (Cell Signaling Technology, # 4370, 1:2000), anti-AKT (Cell Signaling Technology, #4685, 1:1000), anti-P-AKT (Ser473) (Cell Signaling Technology, # 4060, 1:1000), anti-EHF(Proteintech,#27195-1-AP,1:1000), anti-β-Actin (Merck KGaA, #A5441, 1:10000), anti-α-Tubulin (Merck KGaA, #T5168, 1:5000), anti-α-Tubulin (Abcam, #ab18251, 1:5000), Goat-anti-mouse IgG-HRP (Santa Cruz, #sc-2004, 1:20000), Goat-anti-rabbit IgG-HRP (Santa Cruz, #sc-2005, 1:20000). FGFBP2 is expressed as fusion protein with C-terminal tGFP tag and appears as a protein band of about 52 kDa.

### RNA-Seq

RNA samples from PANC-1 cells treated with conditioned media from FGFBP2 wild type (FGFBP2^WT^) and FGFBP2-mutated (FGFBP2^MUT^) expressing cells were sequenced. At least 200 ng per sample was sent for sequencing and quality control was performed using agarose gel electrophoresis, NanoDrop™ and the Agilent 2100 Bioanalyzer to ensure RNA integrity and purity. Fragments per kilobase per million base pairs sequenced (FPKM) were used to estimate gene expression. Read counts from this analysis were used as input for differential gene expression analysis. For RNA-seq analyses, multiple testing correction was performed using the Benjamini–Hochberg false discovery rate (FDR) method, with significance defined as adjusted p value < 0.05.

### Expression and survival analysis

GEPIA2 (http://gepia.cancer-pku.cn/index.html) was used to analyze the expression of ETS homologous factor (EHF) in The Cancer Genome Atlas Program (TCGA) and the Genotype-Tissue Expression (GTEx) datasets [24]. The prognostic significance of EHF in PDAC was assessed through Kaplan-Meier survival analysis. Kaplan-Meier curves, generated using the survival package, were utilized to compare overall survival between groups with high and low EHF expression. PDAC molecular subtypes were classified in a curated cohort of 150 TCGA-PDAC cases selected according to published criteria [25]. The Moffitt 50-gene tumor signature [26], comprising 25 classical and 25 basal-like genes, was loaded using data(“Moffitt_2015”) from the R package SignatureHeatmap. The complete gene list is provided in Supplementary Table 3. Samples were divided into two groups by average-linkage hierarchical clustering based on Pearson correlations of standardized signature-gene expression. Signature scores were calculated as the average standardized expression of the corresponding genes. The group with the higher mean classical-minus-basal-like score was designated classical, and the other basal-like. Within each subtype, patients were divided into EHF-high and EHF-low groups according to the median EHF expression level for overall survival analysis.

### scRNA-seq data integration and cluster annotation analysis

The filtered count matrices were transformed into sparse matrices using the Seurat package (v5.1.0) [27]. Single-cell data integration was carried out using Seurat’s canonical correlation analysis (CCA) method. Cells were filtered based on the following criteria: fewer than 500 genes per cell, more than 25% mitochondrial genes, or fewer than 1000 transcripts per cell. Additionally, genes expressed in fewer than 3 cells were removed from downstream analysis. Raw expression data were normalized using the NormalizeData function and scaled using the ScaleData function. Principal component analysis (PCA) was performed using the RunPCA function with default parameters, and dimensionality reduction was performed using the RunUMAP function. Cell clustering was performed using the FindClusters function with a resolution of 0.5. The FindAllMarkers function was used to identify specific genes for each cell subset, and gene marker signatures were used to define cell identities. For differential analyses of single-cell RNA-seq data, multiple testing correction was performed using the Benjamini–Hochberg FDR method, with adjusted p values < 0.05 considered statistically significant.

### KEGG analysis

Differentially expressed genes (DEGs) were identified using the ‘’FindAllMarkers’’ function in Seurat, with a |log2 FC| > 1 and an adjusted p-value < 0.05 as the threshold for significance. Kyoto Encyclopedia of Genes and Genomes (KEGG) enrichment pathway analysis was performed using the R-package ‘’clusterProfiler’‘ [28].

### Copy Number Variation (CNV) estimation

To detect large-scale chromosomal CNVs, the InferCNV package (version 1.22.0) was used to infer CNVs in ductal cells and identify tumor cells with the default parameters [29]. Macrophages and NK cells served as the reference normal set.

### Cell-cell communication analysis

Cell–cell communication network inference and analysis were performed using CellChat (version 2.1.2) [30] and visualized intercellular communication interactions between cell subsets at the single-cell level.

### Spatial transcriptomics data analysis and cell type annotation

The gene-spot count matrices were analyzed with the Seurat package (v5.1.0) [27]. For each patient slice, normalization across spots was performed using the SCTransform function. Dimensionality reduction was performed using Principal Component Analysis (PCA) and clustering was performed using the FindNeighbors function, followed by identification of cell populations using the FindClusters function. For cell type annotation, robust cell type decomposition (RCTD) was used to deconvolve cell identities within each spot [31].

### Spatial correlation analysis

Spatial correlation analysis was performed as previously described [32], with genes expressed in adjacent spots in ST considered potentially informative. The average normalized gene expression was calculated over a ‘’sliding window’’ of spot groups, consisting of a central spot surrounded by its N nearest neighbors (*N* = 6). This generated a matrix of genes by average expression across all spots. Then, Pearson correlations were used to calculate the relationship between genes.

### Statistical Analysis

Statistical analyses were performed with GraphPad Prism v10 (GraphPad, San Diego, USA), using one-way ANOVA with Tukey’s test for multiple group comparisons and two-tailed unpaired Student’s t-tests for two-group comparisons. A *P*-value < 0.05 was defined as statistically significant.

## Results

### FGFBP2 p.T186S mutation regulates fibroblast activation

**Fig. 1:**
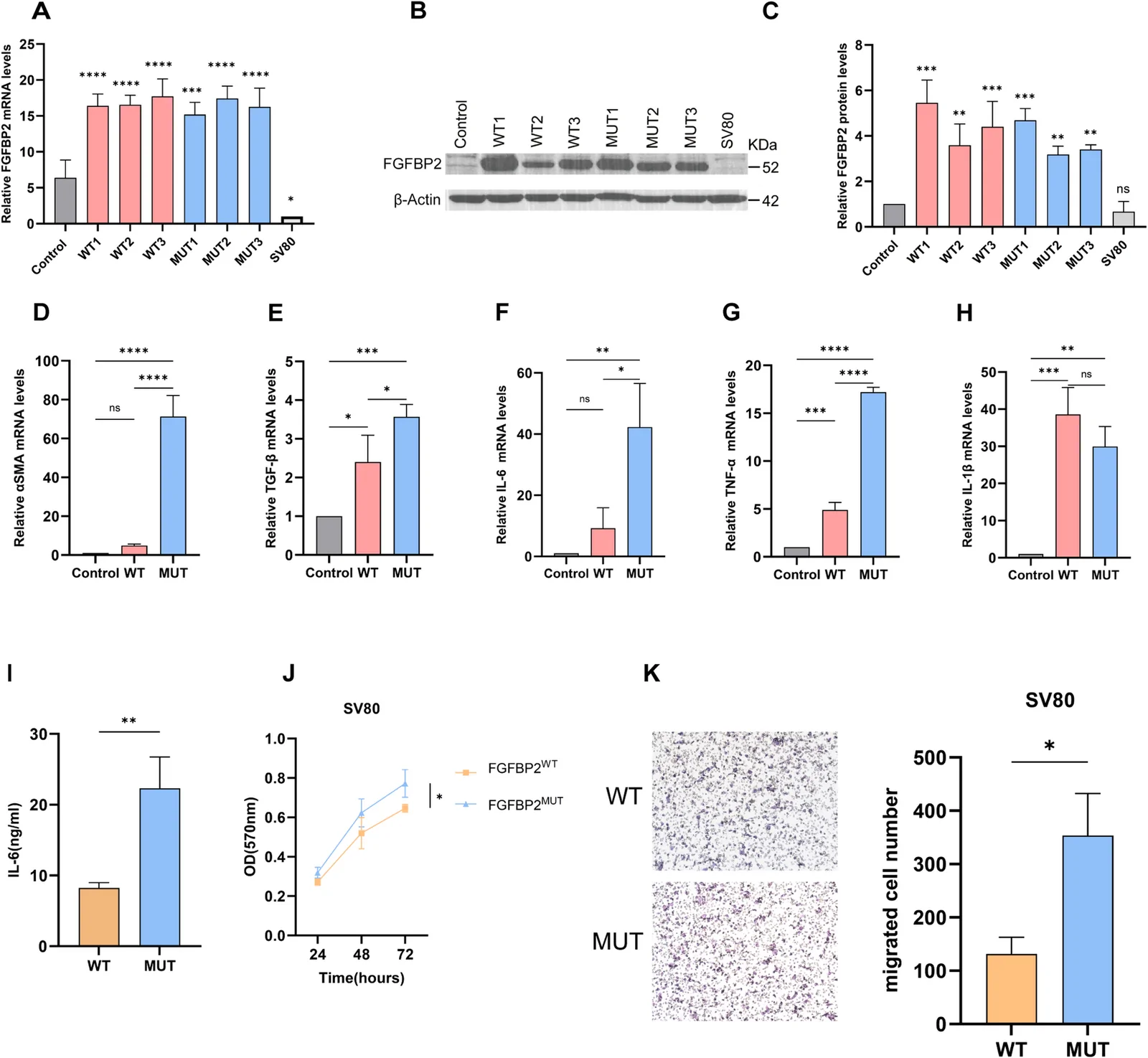
The expression of fibroblast activation markers and cell growth and migration. (**A**) SV80 were transfected with empty (Control), wild type FGFBP2 (WT1, WT2, WT3) and FGFBP2-Mutation (MUT1, MUT2, MUT3) plasmid and verified by qPCR. (**B**,** C**) Western blot images and quantifications of FGFBP2 protein expression in control, wild type and mutant groups. (**D-H**) Expression levels of (**D**) α-SMA, (**E**) TGF-β, (**F**) IL-6, (**G**) TNF-α, (**H**) IL-1β were verified by qPCR in FGFBP2 control, wild type (WT), and mutant (MUT) groups; (**I**) IL-6 levels in conditioned medium derived from FGFBP2 wild type and mutant cells were quantified by ELISA. (**J**) Viable cell numbers of FGFBP2 wild-type (FGFBP2^WT^) and mutant (FGFBP2^MUT^) cells assessed by MTT assay. (**K**) Cell migration of FGFBP2 wild type and mutated cells. (**A-C**,** I-K**) Two-tailed unpaired Student’s t-tests were used for two-group comparisons. (**D-H**) One-way ANOVA followed by Tukey’s post hoc test was used for multiple-group comparisons. Data are presented as mean ± SD from three independent biological replicates, using three independent stable clones.*p < 0.05; **p < 0.01; ***p < 0.001; ns, not significant

To investigate the role of FGFBP2 p.T186S point mutation in SV80 fibroblasts, we transfected cells with an empty plasmid (Control), with a plasmid overexpressing wild type FGFBP2 (FGFBP2^WT^) and with a plasmid overexpressing FGFBP2 p.T186S (FGFBP2^MUT^). QPCR was performed to confirm overexpression of FGFBP2 in FGFBP2^WT^ and FGFBP2^MUT^ as compared to Control (Fig. 1A). Transfection efficacy was further verified at the protein level (Fig. 1B, C). Investigations of α-SMA which is a key marker of fibroblast activation, revealed its upregulation in fibroblasts carrying the FGFBP2-mutation (Fig. 1D). The FGFBP2 mutation was associated with increased expression of proinflammatory cytokines, including IL-6, TNF-α and TGF-β, consistent with an activated fibroblast phenotype, as upregulation of inflammatory cytokines is a recognized feature of activated fibroblasts [33] (Fig. 1E-G). Moreover, ELISA analysis of conditioned media demonstrated significantly elevated IL-6 levels in the mutant group compared with the wild-type group(*p* = 0.0056) (Fig. 1I). These cytokines are critical in shaping the inflammatory tumor microenvironment and driving tumor initiation and progression [34, 35]. In subsequent experiments, Viable cell numbers and migration were assessed, both of which are characteristics of fibroblast activation. FGFBP2^MUT^ cells showed higher viable cell numbers than FGFBP2^WT^ cells during the observation period. Migration assays further demonstrated higher migration rates in FGFBP2^MUT^ cells. Functional assays revealed that the FGFBP2 mutation increased viable cell numbers and enhanced cell motility (*P* = 0.04, *P* = 0.01, respectively; Fig. 1J, K). Taken together, these results indicate that the p.T186S FGFBP2 mutation contributed to fibroblast activation.

### FGFBP2 p.T186S mutation promotes viable cell numbers of pancreatic cancer cells

**Fig. 2:**
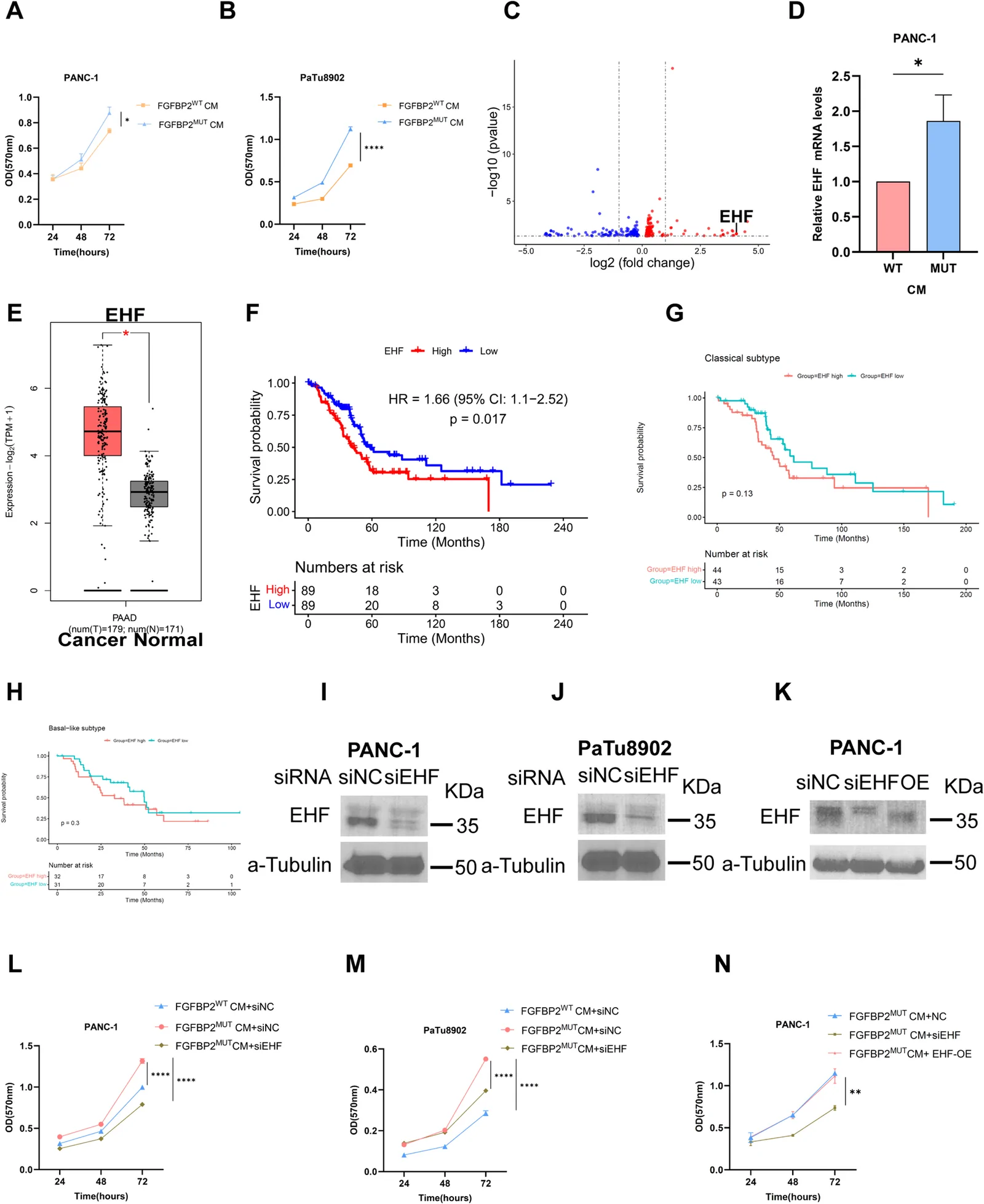
FGFBP2 mutant conditioned medium increases viable cell numbers and induces EHF expression in pancreatic cancer cells. (**A**,** B**) Viable cell numbers of PANC-1 and PaTu 8902 cells treated with conditioned medium (CM) from FGFBP2 wild-type and mutant cells. (**C**) Volcano plot showing altered genes (upregulation, red or downregulation, blue) in PANC-1 cells treated with conditioned medium from FGFBP2 mutated and wild type cells. (**D**) qPCR analysis of EHF expression of PANC-1 cells treated with conditioned medium from FGFBP2 wild type and mutated cells. (**E**) EHF expression in pancreatic cancer and normal tissues. (**F**) Overall survival (OS) analysis patients with high and low expression of EHF. (**G**,** H**) Kaplan–Meier overall survival (OS) curves of patients with high and low EHF expression in the classical and basal-like subtypes, respectively. (**I**,** J**) EHF protein expression in PANC-1 and PaTu 8902 cells after siRNA transfection was examined by immunoblotting. (**K**) EHF protein expression in PANC-1 knockdown cells after EHF overexpression was examined by immunoblotting. (**L**,** M**) Viable cell numbers of PANC-1 and PaTu 8902 cells transfected with siNC or siEHF was assessed after treatment with FGFBP2 wild type or mutant conditioned medium. (**N**) Viable cell numbers of PANC-1 cells transfected with siNC, siEHF, or siEHF followed by EHF overexpression (OE) was assessed after treatment with FGFBP2 mutant conditioned medium. (**A**,** B**,** D**,** L**,** M**) Two-tailed unpaired Student’s t-tests were used for two-group comparisons. (**N**) One-way ANOVA followed by Tukey’s post hoc test was used for multiple-group comparisons. Data are presented as mean ± SD from three independent biological replicates, using three independent stable clones.*p < 0.05; **p < 0.01; ***p < 0.001; ns, not significant

Growth assays on PANC-1and PaTu 8902 cells treated with conditioned media revealed that medium derived from the FGFBP2-mutated fibroblasts increased viable cell numbers of pancreatic cancer cells as compared to controls (Fig. 2A, B). For further mechanistic insights, RNA-Seq analysis was conducted on PANC-1 cells treated with conditioned media. Differential expression analysis revealed that ETS homologous factor (EHF) was upregulated in PANC-1 cells treated with conditioned medium from FGFBP2-mutated fibroblasts (Fig. 2C and Table S2). EHF plays a pivotal and diverse role in the regulation of epithelial cell differentiation, influencing several molecular pathways that control cell proliferation and the maintenance of epithelial homeostasis [36, 37]. The results were validated by measuring EHF expression levels using qPCR (Fig. 2D). The comparison of EHF expression between pancreatic cancer and normal pancreatic tissues, as well as the overall-survival analysis, was performed using publicly available datasets. Comparison of EHF expression between pancreatic cancer and normal pancreatic tissues showed higher EHF expression in tumor tissues. Patients with high EHF expression had shorter median overall survival than those with low EHF expression (median OS: 43.2 months vs. 58.5 months, respectively; Fig. 2E, F). Subtype-specific survival analysis in the curated cohort of 150 TCGA-PDAC cases, classified using the Moffitt basal-like/classical tumor signature, showed no statistically significant difference in overall survival between the EHF-high and EHF-low groups in either the basal-like subtype (*P* = 0.3 or the classical subtype (*P* = 0.13; Fig. 2G, H). To assess whether EHF mediates the pro-proliferative effects of FGFBP2 mutant fibroblast derived conditioned media, EHF was silenced in pancreatic cancer cells using siRNA. Efficient EHF knockdown was confirmed in PANC-1 and PaTu 8902 cells (Fig. 2I, J). EHF re-expression after overexpression was confirmed in PANC-1 knockdown cells (Fig. 2K). Accordingly, under FGFBP2 mutant conditioned media, siEHF transfected cells showed reduced viable cell numbers compared with siNC controls (Fig. 2L, M). Re-expression of EHF restored the viable cell number in PANC-1 knockdown cells (*P* = 0.002, Fig. 2N). Thus, the FGFBP2 mutation in fibroblasts is associated with increased viable cell numbers of pancreatic cancer cells and enhanced EHF expression.

### Pancreatic cancer cells are affected by fibroblast FGFBP2 mutation via ERK1/2 signaling

**Fig. 3:**
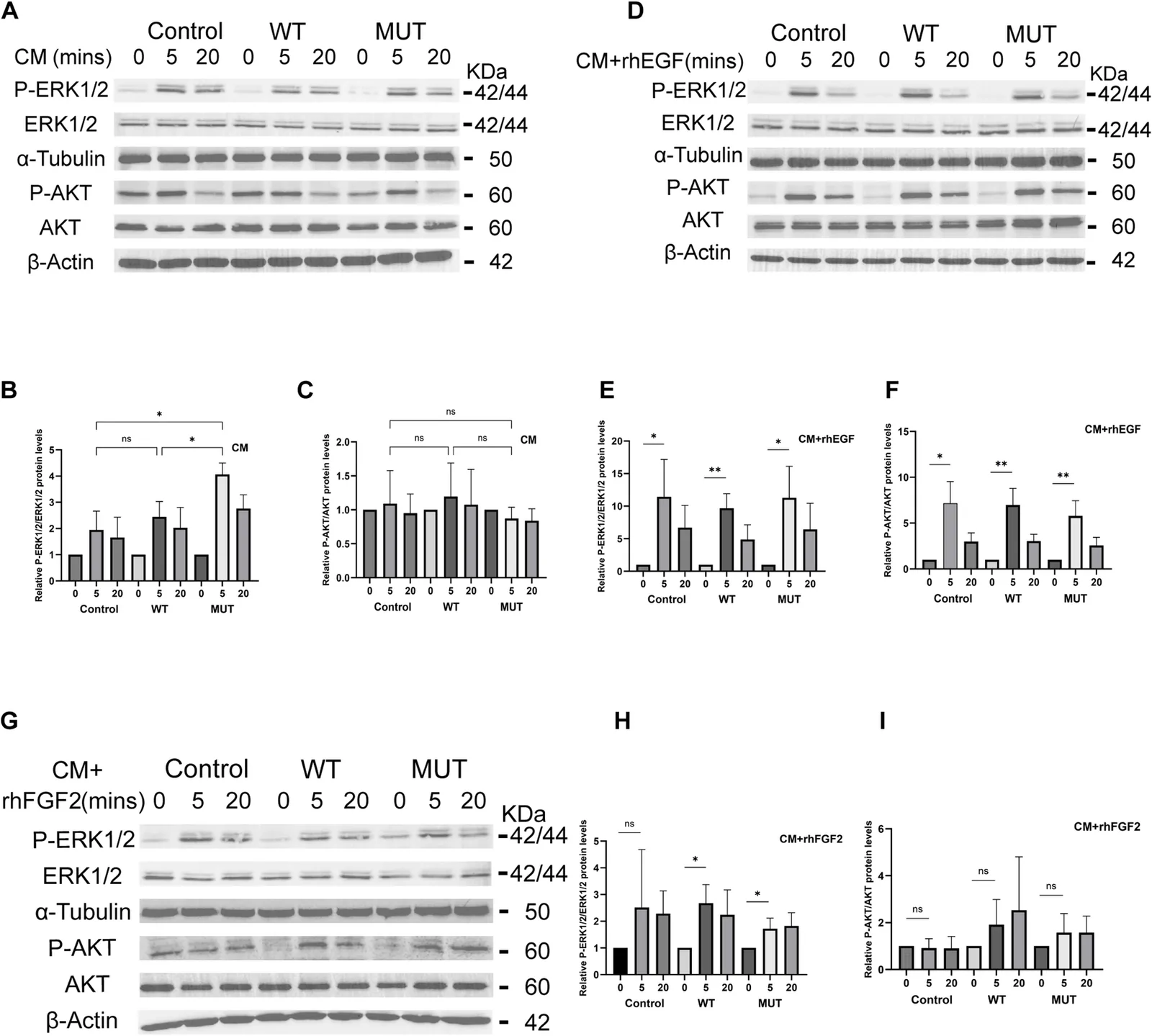
Effects of FGFBP2 wild-type and mutant conditioned media, with or without rhEGF and rhFGF2, on ERK1/2 and AKT signaling in PANC-1 cells. (**A-C**) Western blot for ERK1/2, P-ERK1/2, AKT, and P-AKT in PANC-1 cells treated with conditioned medium of FGFBP2 control (Control), wild type (WT), and mutant cells (MUT). (**D-F**) Western blot for ERK1/2, P-ERK1/2, AKT, and P-AKT in PANC-1 cells treated with conditioned medium of FGFBP2 control (Control), wild type (WT), and mutant cells (MUT) supplemented with rhEGF. (**G-I**) Western blot for ERK1/2, P-ERK1/2, AKT, and P-AKT in PANC-1 cells treated with conditioned medium of FGFBP2 control (Control), wild type (WT), and mutant cells (MUT) supplemented with rhFGF2. Data are presented as mean ± SD from three independent biological replicates; (**B**,** C**,** E**,** F**,** H**,** I**) Two-tailed unpaired Student’s t-tests were used for two-group comparisons. Data are presented as mean ± SD from three independent biological replicates; (B, C, E, F, H, I) results are presented as ratios of phosphorylated and unphosphorylated forms of the proteins; The numbers “0”, “5”, and “20” indicate the stimulation time points (0, 5, and 20 min, respectively). *p < 0.05; **p < 0.01; ***p < 0.001; ns, not significant

**Fig. 4:**
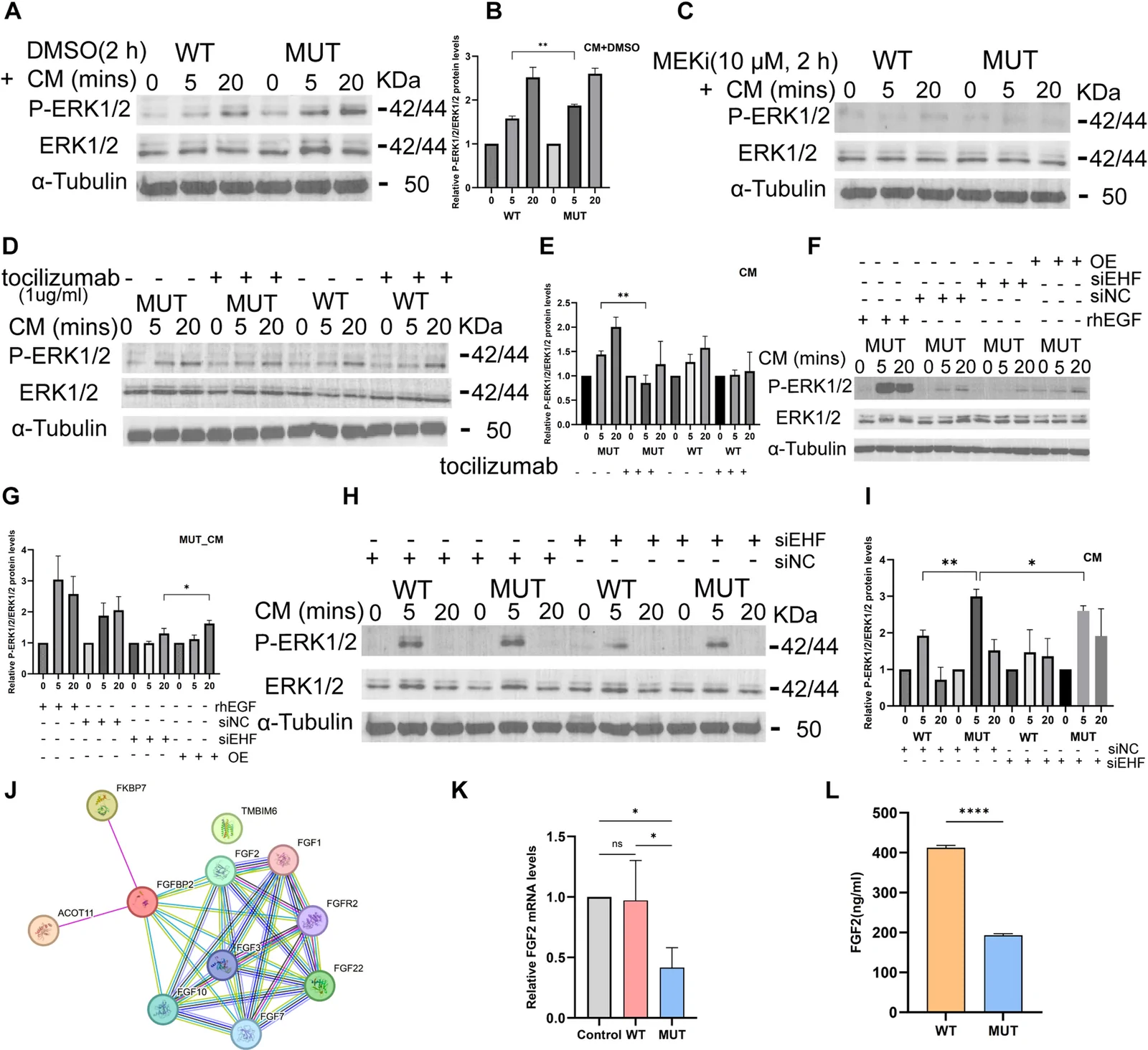
Effects of MEK inhibition, IL-6R blockade, and EHF modulation on ERK1/2 signaling induced by FGFBP2 mutant fibroblast-derived conditioned medium in PANC-1 cells. (**A-C**) Western blot analysis of ERK1/2 and P-ERK1/2 in PANC-1 cells treated with conditioned medium derived from FGFBP2 wild type and mutant cells following pretreatment with DMSO or the MEK inhibitor U0126 (10 µM, 2 h). (**D**,** E**) ERK1/2 phosphorylation in PANC-1 cells treated with FGFBP2 wild-type or mutant conditioned medium in the presence or absence of tocilizumab(1ug/ml,1 h) was examined by immunoblotting. (**F**,** G**) ERK1/2 phosphorylation in PANC-1 cells transfected with siNC, siEHF, or siEHF combined with EHF overexpression plasmid (OE), followed by stimulation with FGFBP2 mutant conditioned medium, was examined by immunoblotting. (**H**,** I**) Western blot analysis of ERK1/2 and P-ERK1/2 in PANC-1 cells transfected with siNC or siEHF and treated with conditioned medium derived from FGFBP2 wild type and mutant cells. (**B**, **E**, **G**, **I**) results are presented as ratios of phosphorylated and unphosphorylated forms of the proteins; (**J**) STRING Analysis of the interaction between FGFBP2 and FGF2. (**K**) FGF2 expression levels were verified by qPCR in FGFBP2 control (Control), wild-type (WT), and mutant cells (MUT); (**L**)FGF2 levels in conditioned medium derived from FGFBP2 wild type and mutant cells were quantified by ELISA. (**B**,** E**,** G**,** I**) Two-tailed unpaired Student’s t-tests were used for two-group comparisons. (**K**) One-way ANOVA followed by Tukey’s post hoc test was used for multiple-group comparisons. Data are presented as mean ± SD from three independent biological replicates. *p < 0.05; **p < 0.01; ***p < 0.001; ns, not significant

FGF2 regulates key biological processes such as cell proliferation and migration by activating FGFR and downstream pathways, including Ras-MAPK (ERK1/2, p38 MAPK, JNK) and PI3K-Akt [16]. To investigate the effect of FGF2 on cancer cells, we cultured PANC-1 cells using conditioned medium (CM) supplemented with rhFGF2 or CM obtained from three groups of FGFBP2-transfected cells, with EGF as a control. Either CM or rhEGF-CM and rhFGF2-CM increased the activation of the canonical ERK1/2 signaling cascade as shown by increased phosphorylation (Fig. 3A, B, D and E and Fig. 3G, H). In addition, rhEGF-CM promoted the activation of the canonical AKT signaling cascade through increased phosphorylation (Fig. 3D, F), whereas CM or rhFGF2-CM did not enhance AKT signaling activation (Fig. 3A, C and Fig. 3G, I). Moreover, CM derived from FGFBP2-mutated cells enhanced ERK1/2 signaling activation via phosphorylation more than that derived from FGFBP2^WT^ cells (MUT vs. WT, *P* = 0.036, Fig. 3A, B). MEK inhibition (10µM, 2 h) suppressed ERK1/2 phosphorylation under conditioned media treatment, confirming MAPK pathway dependence (Fig. 4A-C). IL-6R inhibition with tocilizumab attenuated ERK1/2 phosphorylation induced by FGFBP2 mutant conditioned medium, indicating that IL-6/IL-6R signaling contributes to ERK1/2 activation in response to FGFBP2 mutant conditioned medium (Fig. 4D, E). To assess whether EHF is required for ERK activation, EHF was silenced using siRNA. EHF knockdown reduced ERK1/2 phosphorylation under FGFBP2 mutant conditioned media (Fig. 4H, I). Re-expression of EHF increased ERK1/2 phosphorylation in PANC-1 knockdown cells (Fig. 4F, G). It has been demonstrated that inhibition of ERK1/2 suppresses epithelial-mesenchymal transition (EMT), upregulates cellular senescence markers and activates autophagy in cancer-associated PSCs, which in turn reduces pancreatic cancer cell proliferation [38]. Together, this suggests that FGFBP2 mutation promoted pancreatic cancer progression through increased activation of ERK1/2 signaling.

### Expression of FGF2 is suppressed in fibroblasts carrying FGFBP2-mutation

FGF2 plays a critical role in cell proliferation and cancer development, and it has been shown that FGFBP2 interacts with FGF2 to enhance its biological activity [14]. Using the STRING database, we identified interactions involving FGFBP2, but its function following mutation remains unclear (Fig. 4J). Interestingly, FGF2 expression was also downregulated in fibroblasts expressing mutated FGFBP2 (MUT vs. WT, *P* = 0.043, *P* < 0.0001, respectively; Fig. 4K, L). This suggests that the mutation of FGFBP2 could lead to a reduction in FGF2 expression, hinting at a possible regulatory relationship between the two proteins in the modulation of fibroblasts.

### Expression of EHF in pancreatic cancer cell clusters and its role in the regulation of signaling pathways

**Fig. 5:**
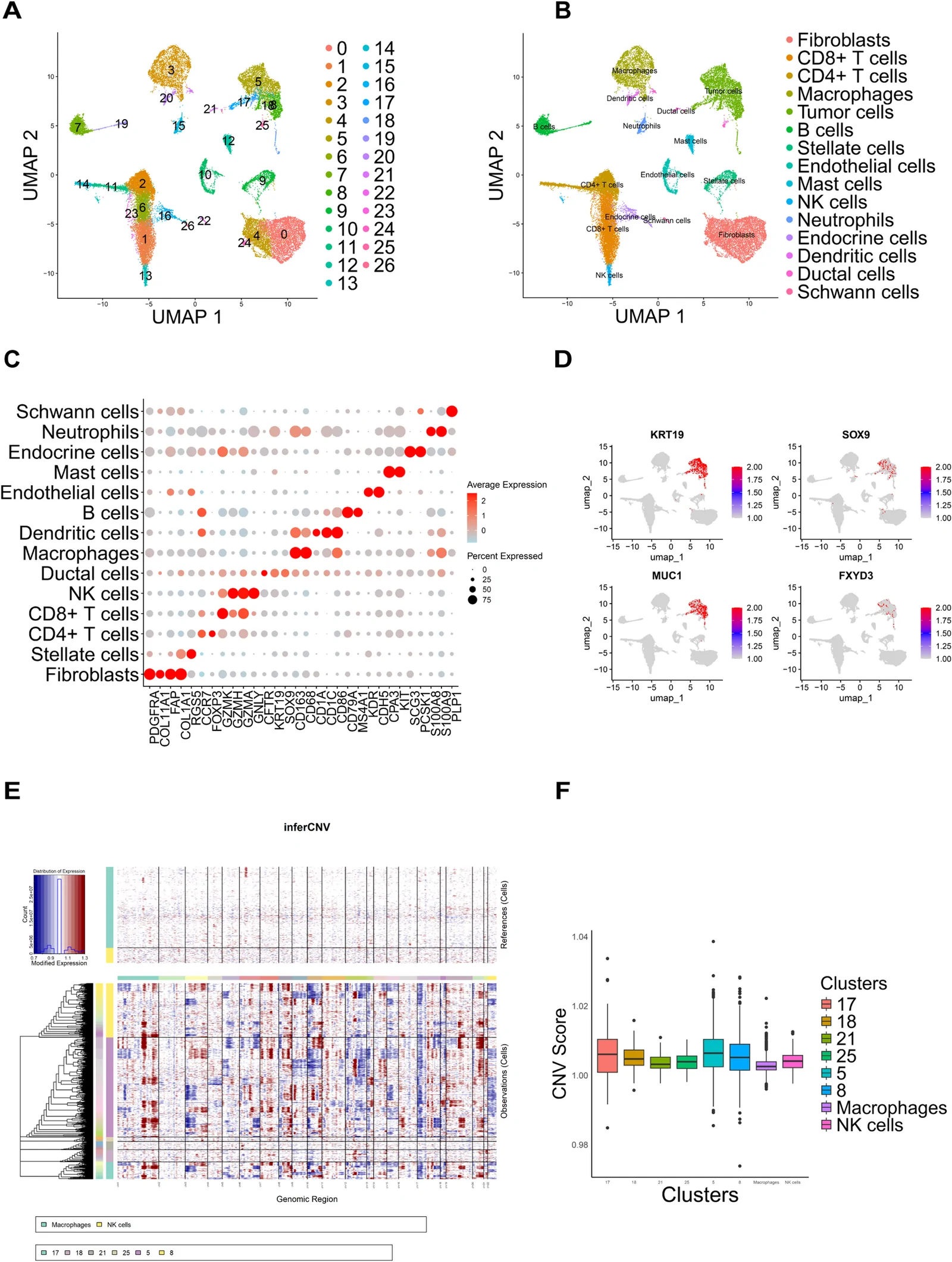
Single-cell RNA sequencing annotation and identification of neoplastic ductal cell clusters. (**A**) Uniform Manifold Approximation and Projection (UMAP) plot displays an integrated cellular map composed of 27 cell clusters. (**B**) UMAP plot displays an integrated cellular map composed of 15 annotated cell types. (**C**) Dot plot showing representative common marker genes in cell types. (**D**) UMAP shows ductal cell markers and cancer cell markers. The expression of FXYD3 and MUC1 was predominantly observed in clusters 5, 8, 17, and 18. (**E**) Heatmap showing ductal cells with different CNV levels. Macrophages and NK cells served as the reference normal set. (**F**) Box Plot showing ductal cells with CNV scores. The ductal cells in clusters 5, 8, 17, and 18 had higher CNV scores compared to macrophages and NK cells that served as the reference normal set

**Fig. 6:**
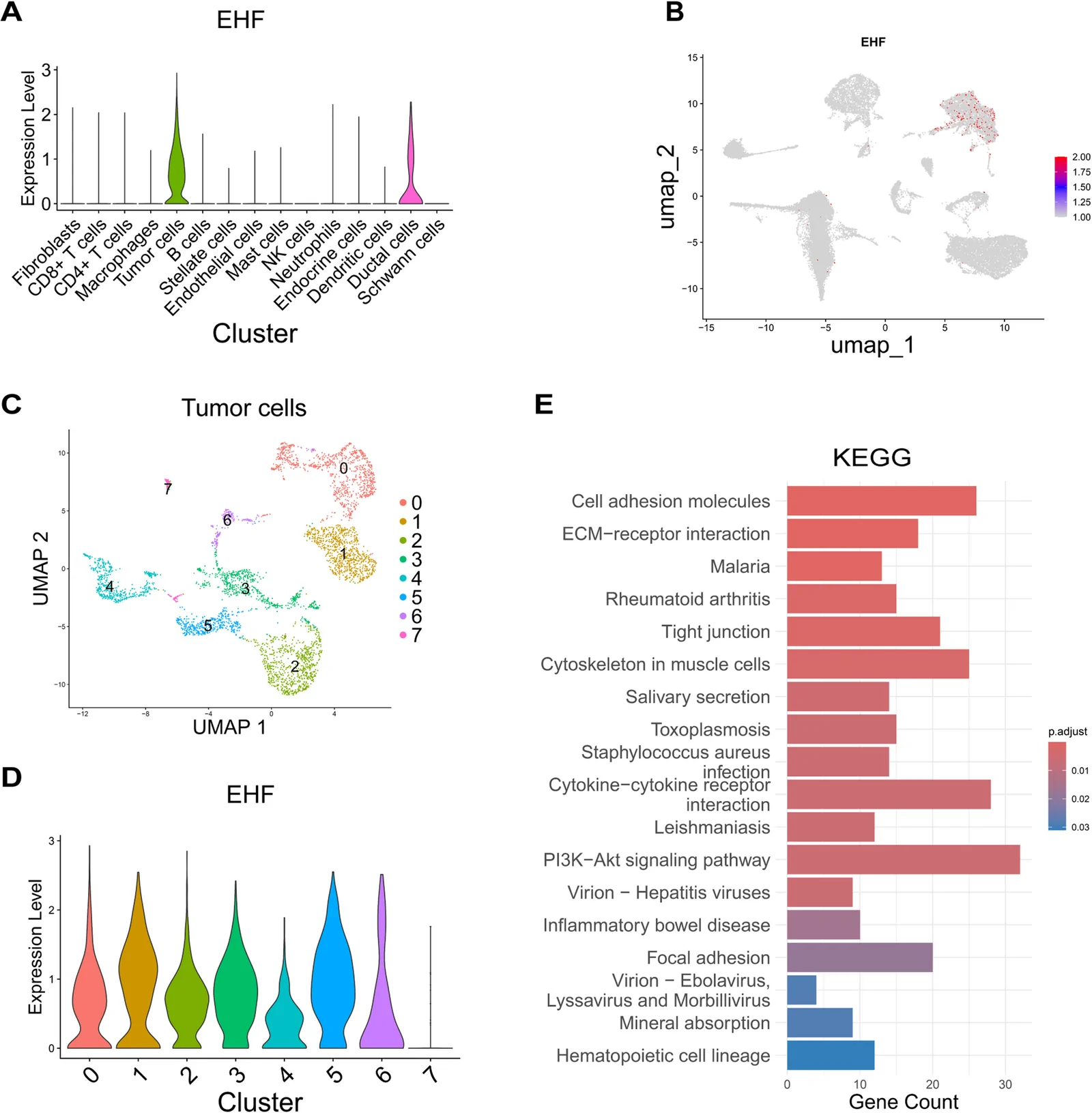
Expression of EHF in different cell types and enrichment analysis. (**A**) VInPlot showing EHF expression in each cell type. (**B**) UMAP showing EHF expression in each cell type. EHF was primarily expressed in tumor cells and ductal cells. (**C**) UMAP showing eight tumor cell clusters. (**D**) VlnPlot showing EHF expression in tumor cell clusters. (**E**) Barplot showing the KEGG enrichment analysis of EHF+ tumor cells and EHF- tumor cells. A positive correlation with PI3K-AKT signaling pathway, ECM-receptor interactions and cell adhesion molecules is noted

As shown above, EHF was upregulated in pancreatic cancer cells treated with conditioned medium from FGFBP2-mutated fibroblasts. To investigate the expression and function of EHF in different cell types, the public single-cell dataset GSE212966 was analyzed [39]. This analysis identified 27 cell clusters (Fig. 5A). The clusters were then annotated using cell cluster-specific marker genes, assigning them to 15 major cell clusters (Fig. 5B). The common marker genes included: fibroblasts (*PDGFRA*,* COL11A1*,* FAP*,* COL1A1*), stellate cells (*RGS5*), CD4 + T cells (*CCR7*,* FOXP3*), CD8 + T cells (*GZMK*,* GZMH*,* GZMA*), NK cells (*GNLY*), ductal cells (*CFTR*,* KRT19*,* SOX9*), macrophages (*CD163*,* CD68*), dendritic cells (*CD1A*,* CD1C*,* CD86*), B cells (*CD79A*,* MS4A1*), endothelial cells (*KDR*,* CDH5*), mast cells (*CPA3*,* KIT*), endocrine cells (*SCG3*,* PCSK1*), neutrophils (*S100A8*,* S100A9*), and Schwann cells (*PLP1*) (Fig. 5C). To identify different cell types within ductal clusters, *KRT19* and *SOX9* were used to represent normal ductal cells, while *FXYD3* and *MUC1* were used to represent neoplastic ductal cells [40, 41]. Furthermore, CNV analysis was used to assess the malignant potential of ductal cell clusters, with macrophages and NK cells serving as the reference normal set. The expression of FXYD3 and MUC1 was predominantly observed in clusters 5, 8, 17, and 18 **(**Fig. 5D). Similarly, CNV analysis revealed that these clusters exhibited higher CNV scores compared to normal cells, leading to their classification as tumor cells (Fig. 5E, F). EHF was primarily expressed in tumor cells and ductal cells, whereas its expression was almost absent in immune and stromal cells (Fig. 6A, B). To analyze EHF expression in tumor cell clusters, these clusters were extracted and divided into 8 clusters (Fig. 6C). Based on the expression levels of the EHF gene, clusters 0, 1, 2, 3, 4, 5, and 6 were defined as EHF+ tumor cells, whereas cluster 7, which shows almost no expression, was defined as EHF- tumor cells (Fig. 6D). KEGG analysis of EHF revealed significant enrichment in the PI3K-AKT signaling pathway, ECM-receptor interactions and cell adhesion molecules (Fig. 6E). These results suggested that EHF might regulate pathways associated with pancreatic cancer cell growth and tumor progression.

### EHF regulates fibroblast signaling through the PDGF pathway

**Fig. 7:**
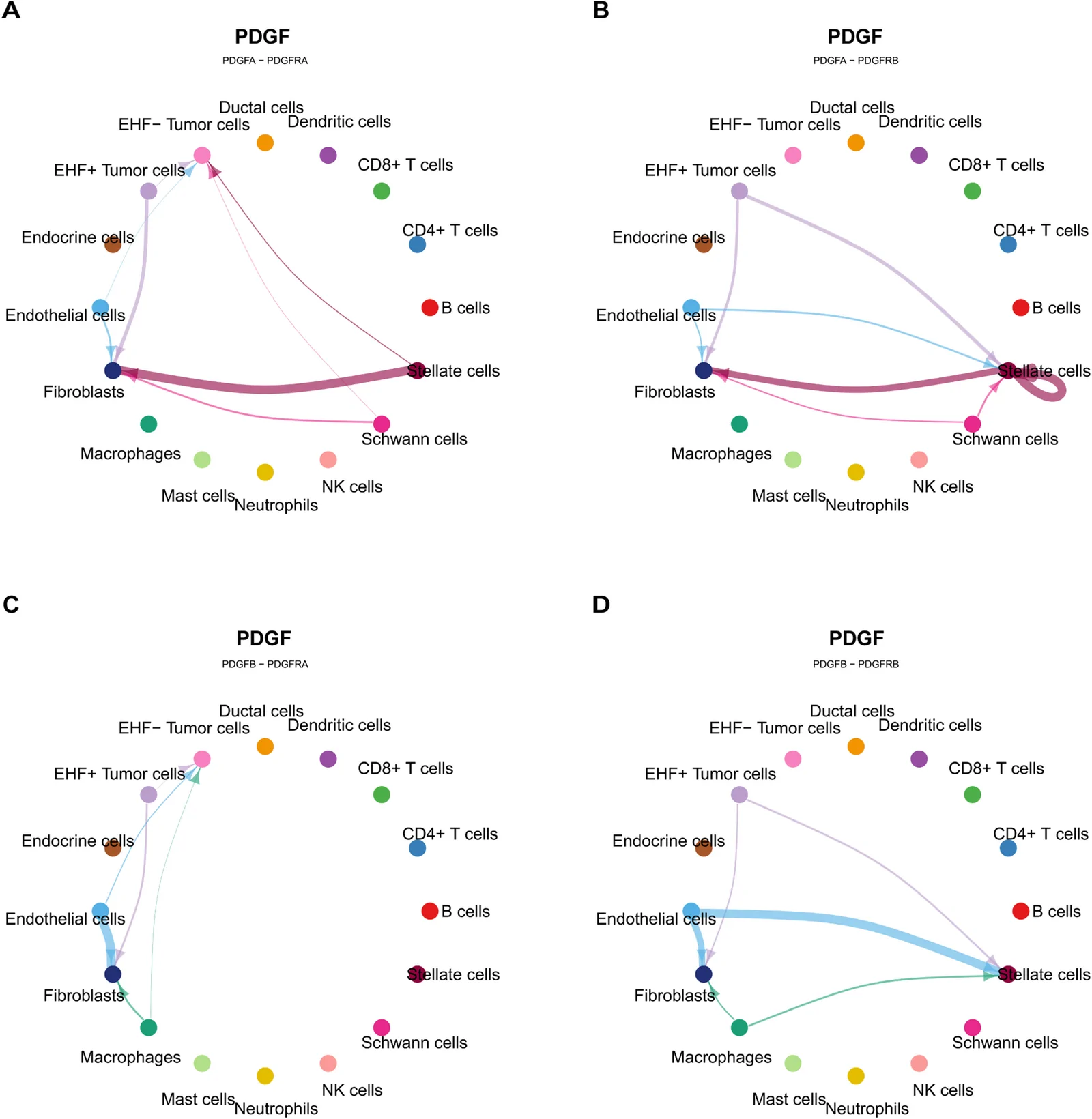
Interaction between the ligands and receptors of the PDGF pathway. (**A**) Circle plot of the cell–cell communication network among PDGF signaling pathway ligand–receptor pairs: PDGFA/PDGFRA. (**B**) Circle plot of the cell–cell communication network among PDGF signaling pathway ligand–receptor pairs: PDGFA/PDGFRB. (**C**) Circle plot of the cell–cell communication network among PDGF signaling pathway ligand–receptor pairs: PDGFB/PDGFRA. (**D**) Circle plot of the cell–cell communication network among PDGF signaling pathway ligand–receptor pairs: PDGFB/PDGFRB. Interaction of the PDGF signaling pathway is observed in EHF+ tumor cells and fibroblasts, whereas no interaction occurred in EHF- tumor cells

**Fig. 8:**
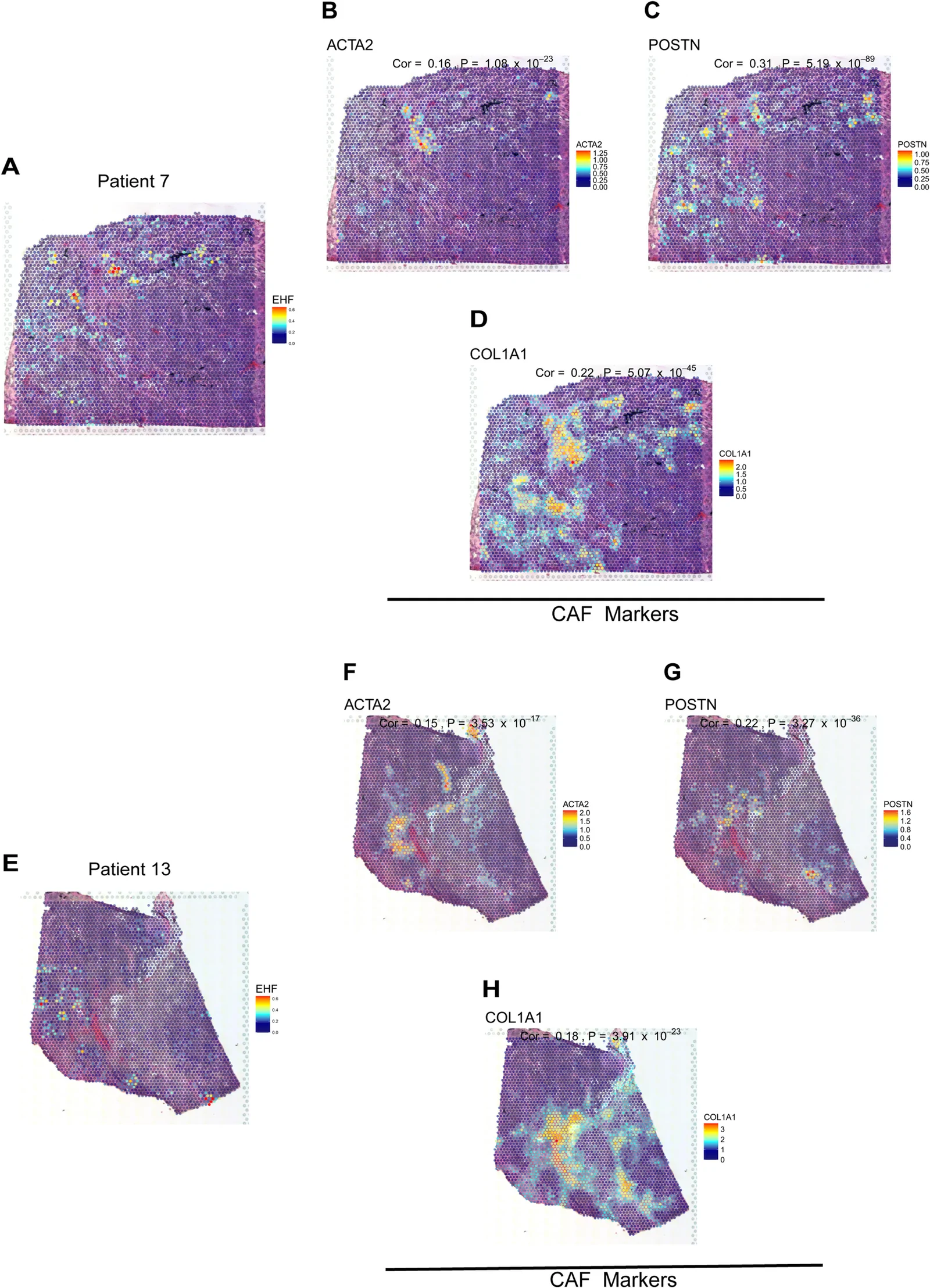
Spatial plots show the correlation between EHF and CAF markers. **(A)** Spatial plots show the spatial distribution of EHF expression from patient 7. (**B-D**) Spatial plots show the co-localization of EHF with CAF markers (ACTA2, POSTN, COL1A1) from patient 7. (**E**) Spatial plots show the spatial distribution of EHF expression from patient 13. (**F-H**) Spatial plots show the co-localization of EHF with CAF markers (ACTA2, POSTN, COL1A1) from patient 13

To further explore the role of EHF in pancreatic cancer and fibroblasts, CellChat was used to analyze the intercellular signaling pathways and ligand-receptor interactions (Fig. S1A-C). In the platelet-derived growth factor (PDGF) signaling pathway, EHF+ tumor cells regulated fibroblasts through PDGF/PDGFR interactions (Fig. 7A-D and Fig. S1B). EHF primarily drove its effects through interactions between PDGF alpha (PDGFA)/PDGF beta (PDGFB) ligands in tumor cells and PDGFRB receptors in fibroblasts (Fig. 7B, D). PDGFA/B are expressed in a broad range of cell types and utilize autocrine or paracrine mechanisms to perform their biological functions [42, 43]. Tumor cells released paracrine signals, including transforming growth factor (TGF-β) and PDGF, which activated fibroblasts [44]. PDGFRα and PDGFRβ, predominantly expressed in fibroblasts, serve as key markers for these cells [45]. Moreover, PDGFRα controlled the fibroblast-to-myofibroblast transition during wound healing [46]. Next, spatial transcriptomic analysis was further conducted to characterize and visualize the spatial distribution of EHF and CAFs. First, by applying dimensionality reduction clustering to the PDAC spatial transcriptomic dataset, several distinct spatial clusters were identified, and fibroblast markers were visualized (Fig. S2, S3, S4). Significant spatial correlation was observed between CAFs markers (*ACTA2*,* POSTN*,* COL1A1*) and EHF in spatial transcriptomics (r range = 0.15–0.31; all *p* < 0.001) (Fig. 8B-D and F-H). This is consistent with the results of the single-cell analysis, despite considerable spatial regional heterogeneity. Altogether, these results suggested that EHF might have driven fibroblast remodeling of the tumor microenvironment by modulating PDGF signaling.

## Discussion

The poor prognosis of PDAC can in part be attributed to the intricate interactions between molecular and cellular components within the TME. CAFs play a critical role in the tumor microenvironment by modulating pro-tumorigenic processes and secreting various cytokines. Here we analyze the role of a specific, previously identified FGFBP2 mutation in facilitating fibroblast activation and promoting tumor progression. Fibroblast activation is influenced by a variety of factors, including mechanical stress, inflammatory signals, and interactions with other cell types in the microenvironment [47]. Quiescent pancreatic stellate cells (PSCs) show increased expression of the CAF-like surface marker α-SMA upon activation by TGF-β, resulting in their conversion to activated CAFs [48]. Similarly, the FGFBP2 mutation in fibroblasts leads to upregulated expression of α-SMA, and enhanced viable cell numbers and migration, reflecting characteristics consistent with those of activated fibroblasts, further supporting their transition to activated CAFs. Additionally, activated fibroblasts produce extracellular matrix (ECM) components and secrete a wide range of cytokines, including TGF-β, TNF-α, IL-6, and IL-1β [9, 49, 50]. Our results indicate that the identified FGFBP2 mutation in fibroblasts induces upregulation of the expression of TGF-β, TNF-α, and IL-6.

The crosstalk between CAFs and tumor cells mainly takes place through paracrine signaling, where CAFs secrete various growth factors and cytokines, such as FGF2, EGF and TGF-β, which effectively promote tumor cell proliferation in vitro [45]. We treated PANC-1 and PaTu 8902 cells with conditioned media and observed that medium derived from FGFBP2-mutated fibroblasts enhanced viable cell numbers of PANC-1 and PaTu 8902 cells, suggesting that this mutation promoted tumor cell growth in a paracrine manner. Furthermore, RNA-Seq analysis of PANC-1 cells treated with conditioned medium revealed that FGFBP2-mutated fibroblasts induced transcriptional changes associated with tumor cell growth. In addition, we found that EHF expression was upregulated in cancer cells treated with conditioned medium from FGFBP2-mutated fibroblasts. EHF is a member of the ETS family of transcription factors, which regulate key processes such as differentiation, proliferation and epithelial homeostasis, and play diverse roles in cancer progression and the tumor microenvironment. In colorectal cancer, EHF activates TGF-β signaling leading to tumor progression [51]. In further analyses EHF was highly expressed in pancreatic cancer tissues and associated with poorer prognosis, and single cell data further supported that EHF was predominantly expressed in pancreatic cancer cells. Although TCGA bulk transcriptomic data represent the combined expression profiles of different cellular compartments, the cellular origin of the association between EHF expression and overall survival cannot be determined from these data. Moreover, because the single-cell and TCGA analyses were derived from independent datasets, the predominant expression of EHF in malignant epithelial cells observed in the single-cell dataset cannot establish the cellular source of the prognostic association observed in TCGA. Therefore, the cellular origin of the bulk EHF survival association remains unresolved. Previous studies have suggested context-dependent functions of EHF in PDAC. In epithelial tumor cells, EHF has been reported to exhibit tumor-suppressive and immune-modulatory properties and was associated with response to PD-1 blockade [52]. In contrast, stromal ESE3/EHF-related signaling has been implicated in tumor-promoting phenotypes. These apparently divergent findings suggest that the biological effects of EHF may depend on its cellular context and interaction with the tumor microenvironment. KEGG analysis indicated that EHF regulated the PI3K-AKT signaling pathway, ECM-receptor interactions and cell adhesion molecules. Moreover, under FGFBP2 mutant conditioned media, pancreatic cancer cells with EHF knockdown showed reduced viable cell numbers. Therefore, FGFBP2 mutation in fibroblasts may lead to the upregulation of EHF in tumor cells, contributing to cancer cell progression.

Tumor cells may further promote fibroblast activation. Chen et al. reported that the loss of annexin A1 (ANXA1) expression in epithelial cells during the progression of esophageal squamous cell carcinoma (ESCC) disrupted fibroblast homeostasis, which led to the transformation of normal fibroblasts into CAFs [53]. Similar studies reported that EHF expression in pancreatic stellate cells (PSCs) influenced PDAC chemoresistance and tumor growth by activating PSCs through the transcription of α-SMA, collagen-I, and IL-1β [54]. Our results show that EHF regulated fibroblast signaling through the PDGF pathway, which influenced key cellular processes such as fibroblast viable cell numbers and migration [55]. EHF expressed in cancer cells may modulate fibroblast function through interaction with PDGF receptors.

FGFBP2 enhances FGF2 signaling by releasing FGF2 from the extracellular matrix, thereby promoting its biological activity [14]. Through STRING data analysis, the direct association between FGFBP2 and FGF2 suggested a functional interaction, which potentially enhances its signaling activity. Moreover, CAFs secreted cytokines that were taken up by cancer cells, promoted effective proliferation in vitro by triggering critical signaling pathways, such as MAPK, through factors like IL-6, FGF and TGF-β [45]. FGF2 bound to its receptor (FGFR), leads to the activation of the MAPK/ERK cascade, which promotes gene expression changes essential for growth and development [16]. Our investigations with conditioned medium revealed that ERK1/2 phosphorylation was enhanced in PANC-1 cells treated with medium derived from FGFBP2-mutated fibroblasts. This suggested that cytokines secreted by FGFBP2-mutated fibroblasts alter cancer cell behavior through increased ERK1/2 phosphorylation. Although FGF2 expression was downregulated in the FGFBP2-mutated fibroblasts, other cytokines may promote the observed changes in ERK1/2 phosphorylation. Ando et al. showed that eicosatetraenoic acid (EPA) reduced cancer angiogenesis in colon CAFs by inhibiting IL-6 and VEGF secretion and ERK phosphorylation [56]. Consistent with a potential role of IL-6/ERK signaling, our data show increased IL-6 levels in conditioned media derived from FGFBP2 mutant fibroblasts. Furthermore, treatment with the IL-6R inhibitor tocilizumab reduced ERK1/2 phosphorylation induced by FGFBP2 mutant conditioned medium, suggesting that IL-6/IL-6R signaling contributes to, but may not exclusively mediate, ERK1/2 activation. Recent evidence further supports the role of fibroblast-derived IL-6 as a biologically active paracrine mediator in pancreatic cancer [57].

Although we found that the FGFBP2 mutation results in EHF upregulation, one of the limitations of the study is that the exact molecular mechanisms are not known and require further investigation. RNA-seq was performed only in PANC-1 cells; therefore, whether the EHF-associated transcriptional response is shared by PaTu 8902 cells remains to be established. Furthermore, the presence and functional consequences of FGFBP2 p.T186S in patient CAFs were not directly validated in tumor specimens, limiting the clinical interpretation of our findings. In addition, the effects of the FGFBP2 mutation on other signaling pathways and on cytokines that affect ERK1/2 signaling were not investigated, which could provide further insight into pancreatic cancer cell progression.

In conclusion, our results demonstrate that the previously identified FGFBP2 mutation promotes fibroblast activation and increases the viable cell population of pancreatic cancer cells. Mechanistically, this effect may be mediated by upregulation of EHF in pancreatic cancer cells. Furthermore, the FGFBP2 mutation in fibroblasts appears to influence cancer cells by modulating ERK1/2 phosphorylation levels. Together, a specific mutation in the non-neoplastic cells of the TME that drives the crosstalk between tumor cells and fibroblasts, thereby promoting pancreatic cancer progression.

## Supplementary Information

Below is the link to the electronic supplementary material.

Supplementary Material 1

Supplementary Material 2

## Data Availability

Published scRNA-seq data on human PDAC from GSE212966 [39] and spatial transcriptomics data from GSE277783 [58] were used in this study. In addition, the datasets analyzed are available from The Cancer Genome Atlas (TCGA) repository (https://portal.gdc.cancer.gov/). The RNA-seq data from this study have been deposited in the NCBI Gene Expression Omnibus (GEO) under accession number GSE318648. The source code used in this study is available at: https://github.com/wxlabbioinfo/FGFBP2-paper.
