# Supplementary material for: FGFBP2 p.T186S mutation in tumor associated fibroblasts supports progression of pancreatic cancer through autocrine and paracrine mechanisms
Source: J Transl Med. 2026 Nov 10;24:1421. doi: 10.1186/s12967-026-09137-3 (PMC13757086; doi:10.1186/s12967-026-09137-3)
Supplement: Supplementary file 1 [file 12967_2026_9137_MOESM2_ESM.pdf]

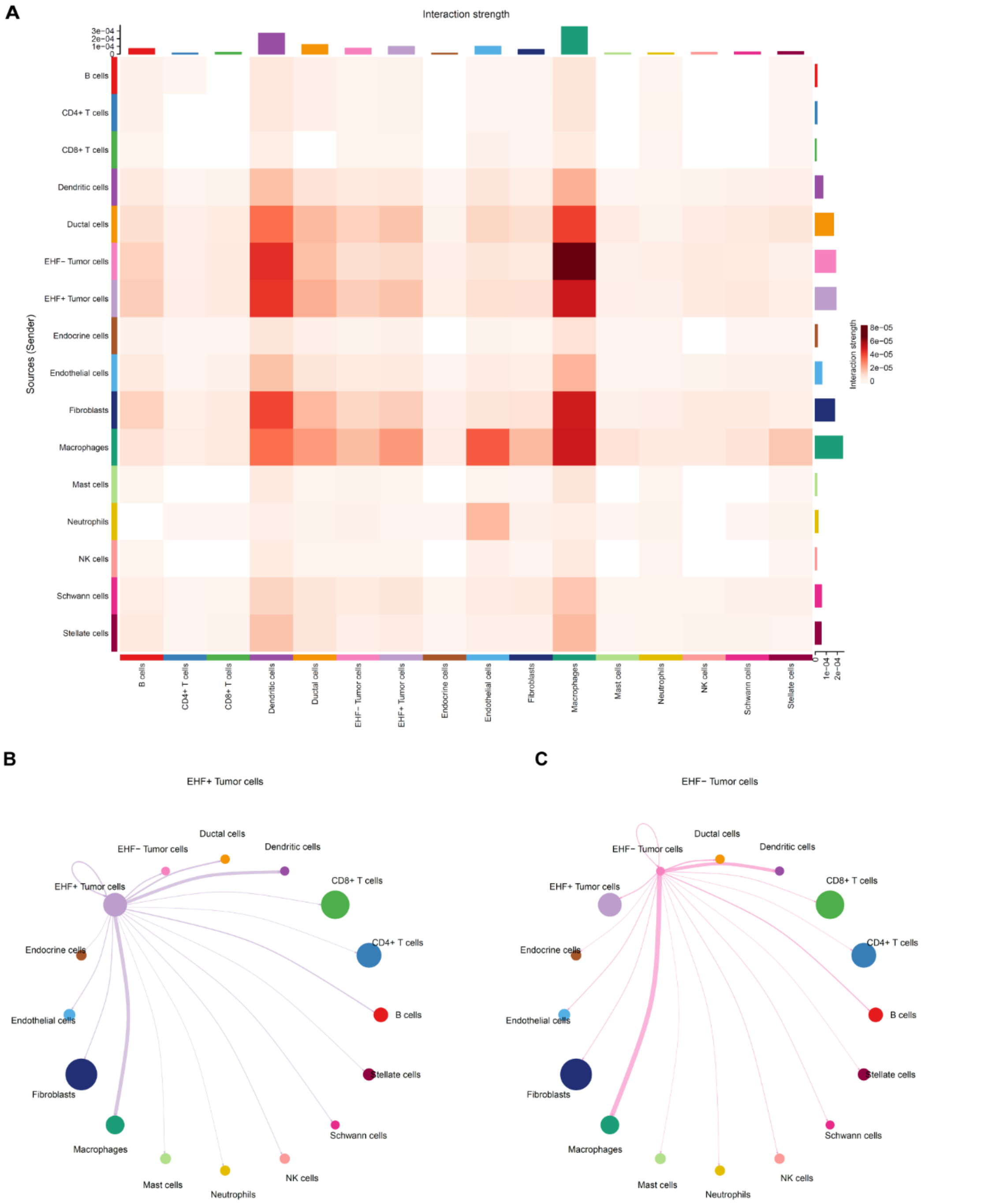

Supplementary figure 1. Cell-cell interaction in major cell types. (**A**) Heatmap of cell-cell interaction strength networks among different cell types. (**B**) Circle plot of the cell–cell communication network among EHF+ tumor cells and other cell types, with edge widths representing the strength of the interactions. (**C**) Circle plot of the cell–cell communication network among EHF- tumor cells, and other cell types, with edge widths representing the strength of the interactions.

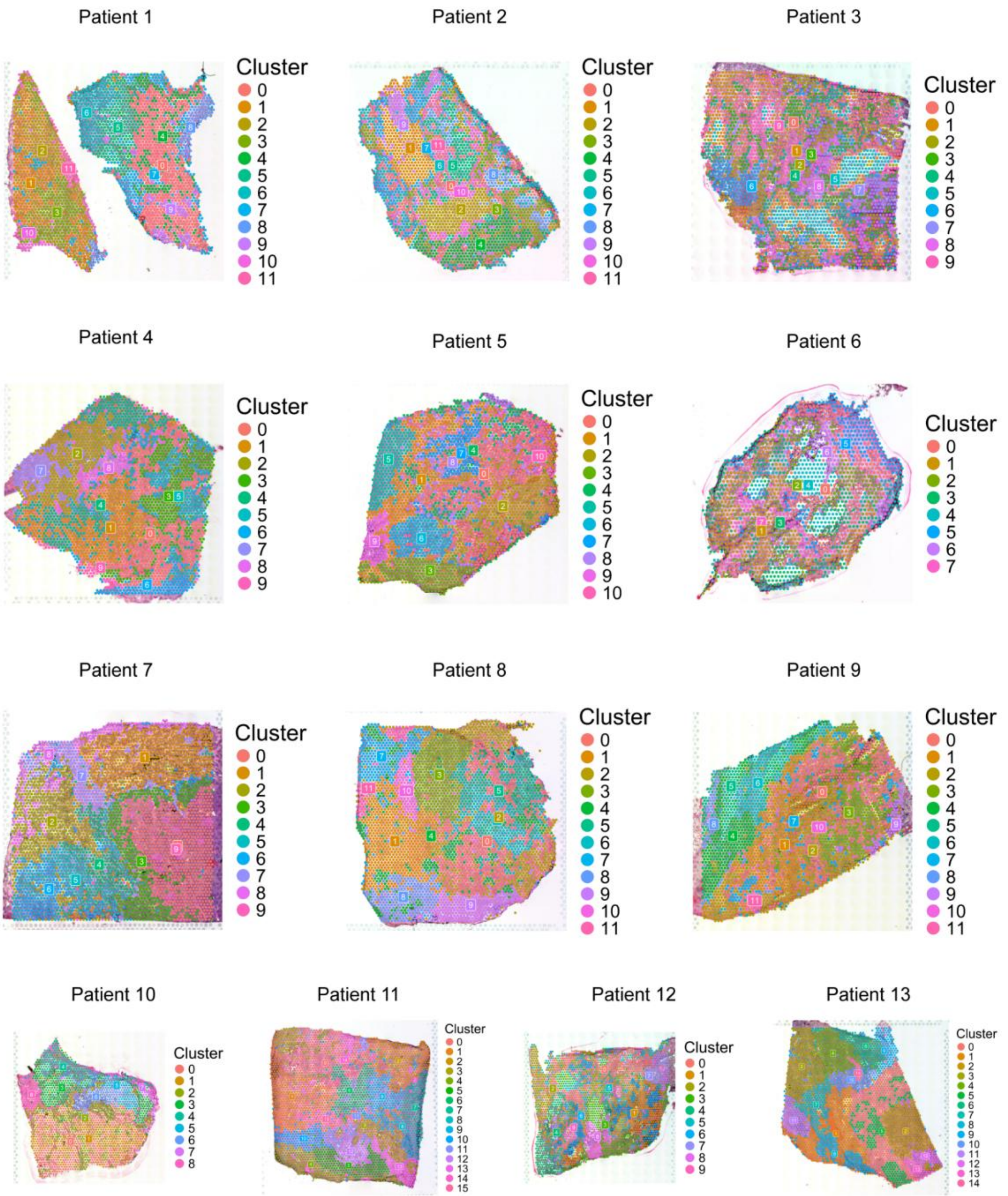

Supplementary figure 2. Spatial cluster map. Spatial transcriptomics (ST) clustering map derived from PDAC tissue samples from 13 patients.

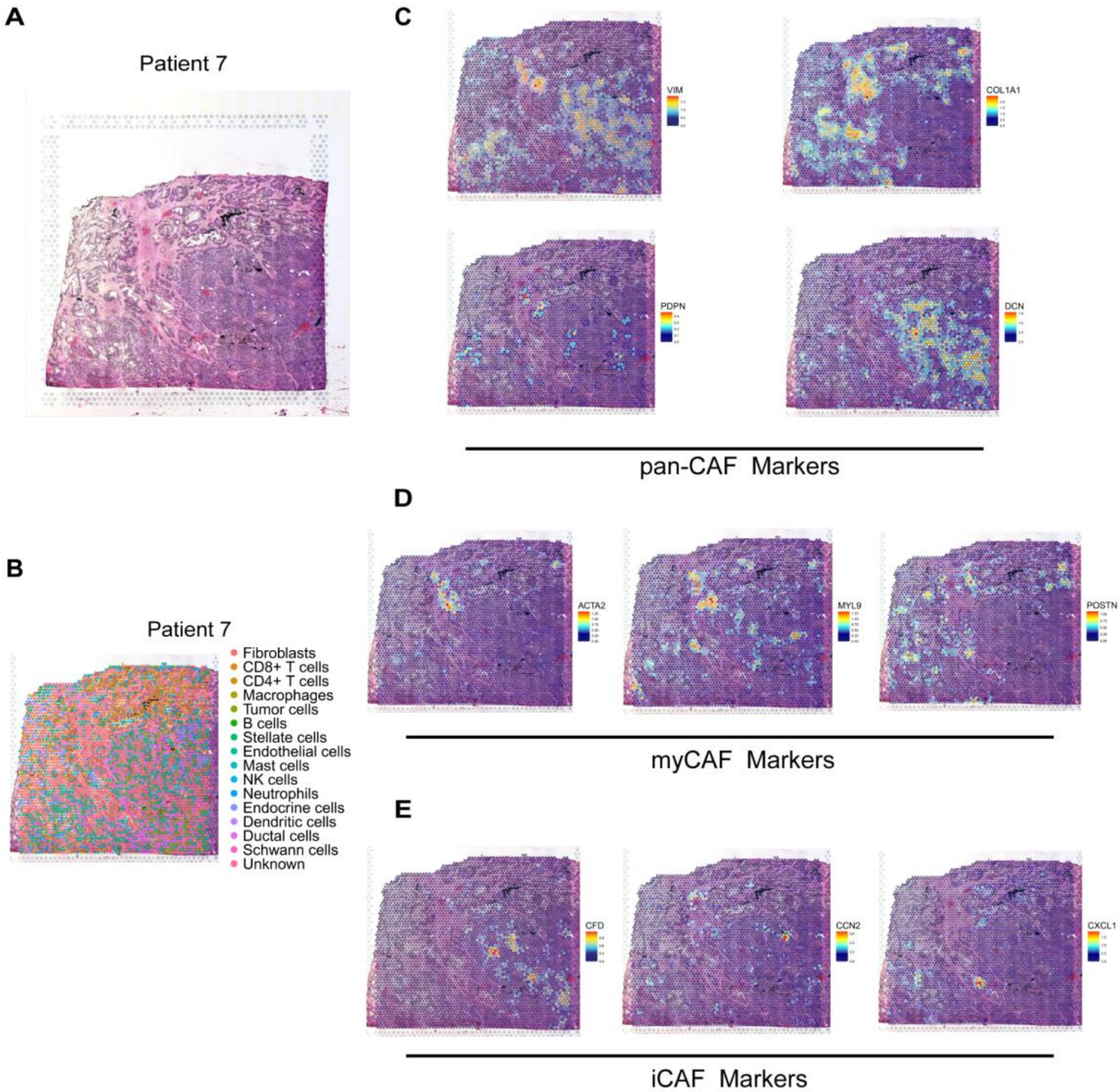

Supplementary figure 3. Spatial position of CAF subset markers.

(**A**) H&E staining of PDAC tissue slices from patient 7. (**B**) Spatial plot showing the cell types identified by RCTD from patient 7. (**C**) Spatial plots show the spatial position of pan-CAF markers (VIM, COL1A1, PDPN, DCN). (**D**) Spatial plots show the spatial position of myCAFs markers (ACTA2,MYL9,POSTN). (E) Spatial plots show the spatial position of iCAFs markers (CFD,CCN2,CXCL1).

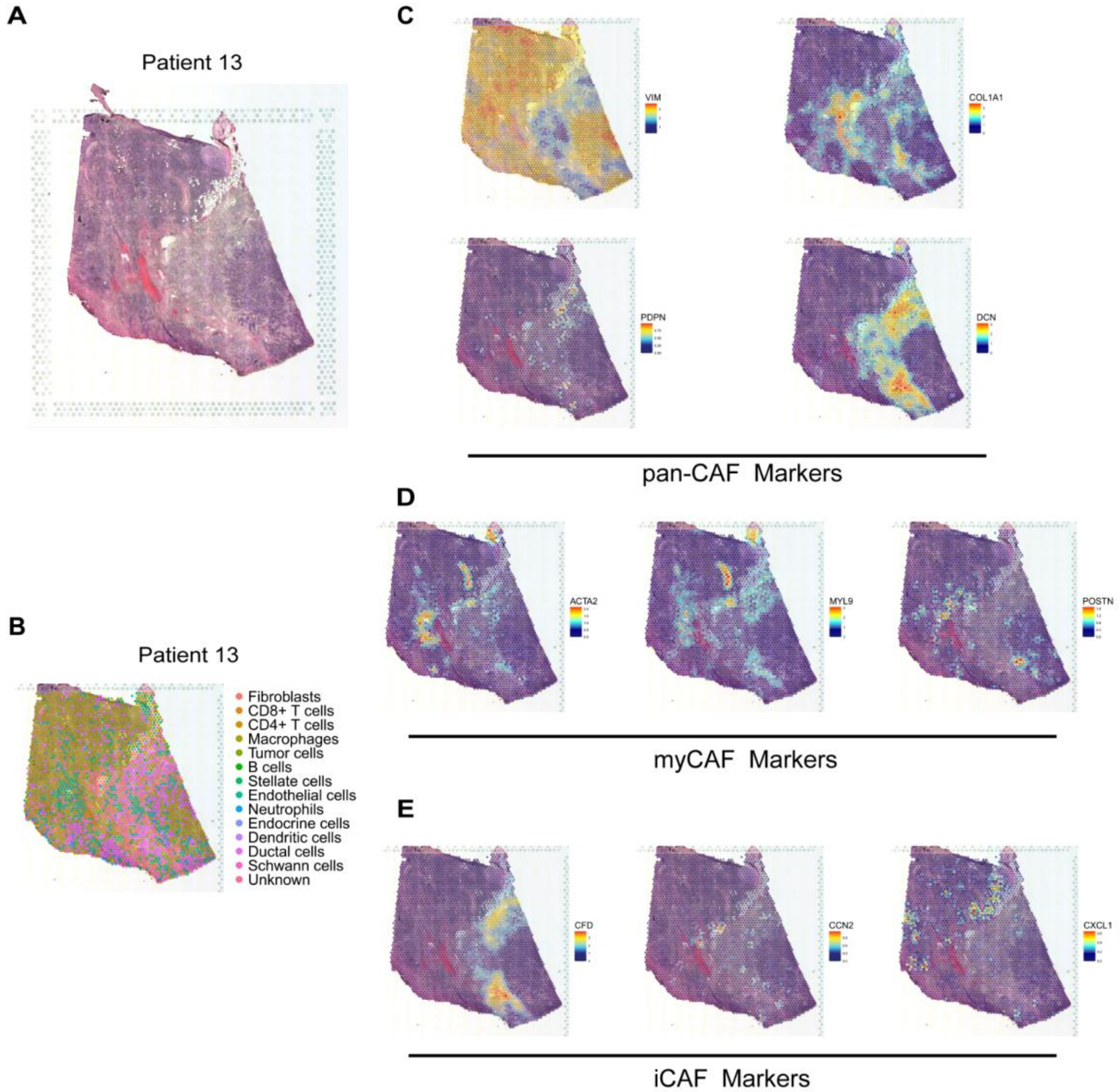

Supplementary figure 4. Spatial position of CAF subset markers.

(**A**) H&E staining of PDAC tissue slices from patient 13. (**B**) Spatial plot showing the cell types identified by RCTD from patient 13. (**C**) Spatial plots show the spatial position of pan-CAF markers (VIM, COL1A1, PDPN, DCN). (**D**) Spatial plots show the spatial position of myCAFs markers (ACTA2, MYL9,POSTN). (E) Spatial plots show the spatial position of iCAFs markers (CFD, CCN2,CXCL1).

Supplementary table 1. human primers

gene	Forward primer (5’ → 3’)	Reverse primer (5’ → 3’)
FGFBP2	CTG CTG ACC CCA AAC CTT ACT	CCT TGA GGC TGG AAG TCA CC
α-SMA	CTA TGC CTC TGG ACG CAC AAC T	CAG ATC CAG ACG CAT GAT GGC A
TGF-β	TAC CTG AAC CCG TGT TGC TCT C	GTT GCT GAG GTA TCG CCA GGA A
IL-1β	CCA CAG ACC TTC CAG GAG AA	GTG CAG TTC AGT GAT CGT ACA GG
IL-6	AGA CAG CCA CTC ACC TCT TCA G	TTC TGC CAG TGC CTC TTT GCT G
TNF-α	CTC TTC TGC CTG CTG CAC TTT G	ATG GGC TAC AGG CTT GTC ACT C
FGF2	AGC GGC TGT ACT GCA AAA ACG G	CCT TTG ATA GAC ACA ACT CCT CTC
EHF	ATC AGA GGC AGT GGC TCA GCT A	ACC AGT CTT CGT CCA TCC ACA C
β-Actin	AGG CAC CAG GGC GTG AT	GCC CACA TA GGA ATC CTT CTG AC

Supplementary table 2. List of the top 20 differentially expressed genes in PANC-1 cells treated with conditioned medium from FGFBP2 mutated cells (in comparison to PANC-1 cells treated with conditioned medium from FGFBP2 wild type cells)

Genes	Log2 FC	P value
BOLA2B	4.524	0.001
AC073592.6	4.417	0.017
SOX7	4.057	0.028
AC008073.1	4.056	0.030
EHF	4.056	0.030
AL121829.1	3.945	0.041
BX255925.2	3.898	0.015
RPL10P19	3.897	0.043
C9orf131	3.869	0.013
MT-TK	3.675	0.024
RN7SL473P	3.489	0.034
AC008758.4	3.477	0.035
C4orf54	3.476	0.037
MT1JP	3.440	0.013
TATDN1P1	3.375	0.049
AC007881.3	3.119	0.004
AC007216.2	2.862	0.008
AL627230.3	2.662	0.047
HRAT5	2.365	0.032
RBMS2P1	1.845	0.014
